# Robot Guidance Using Machine Vision Techniques in Industrial Environments: A Comparative Review

**DOI:** 10.3390/s16030335

**Published:** 2016-03-05

**Authors:** Luis Pérez, Íñigo Rodríguez, Nuria Rodríguez, Rubén Usamentiaga, Daniel F. García

**Affiliations:** 1 Fundación PRODINTEC, Avda. Jardín Botánico 1345, 33203 Gijón (Asturias), Spain; irf@prodintec.com (I.R.); nrl@prodintec.com (N.R.); 2 Department of Computer Science and Engineering, Universidad de Oviedo, Campus de Viesques, 33203 Gijón (Asturias), Spain; rusamentiaga@uniovi.es (R.U.); dfgarcia@uniovi.es (D.F.G.)

**Keywords:** machine vision, 3D sensors, perception for manipulation, robot guidance, robot pose, part localization

## Abstract

In the factory of the future, most of the operations will be done by autonomous robots that need visual feedback to move around the working space avoiding obstacles, to work collaboratively with humans, to identify and locate the working parts, to complete the information provided by other sensors to improve their positioning accuracy, *etc*. Different vision techniques, such as photogrammetry, stereo vision, structured light, time of flight and laser triangulation, among others, are widely used for inspection and quality control processes in the industry and now for robot guidance. Choosing which type of vision system to use is highly dependent on the parts that need to be located or measured. Thus, in this paper a comparative review of different machine vision techniques for robot guidance is presented. This work analyzes accuracy, range and weight of the sensors, safety, processing time and environmental influences. Researchers and developers can take it as a background information for their future works.

## 1. Introduction

Since the end of the 18th century with the first Industrial Revolution through the introduction of mechanical production facilities powered by water and steam, factories have experimented big changes in their production systems [1]. The second Industrial Revolution, in the start of the 20th Century, introduced mass production based on the division of labor powered by electrical energy [2]. The third Industrial Revolution of the start of 1970s introduced the use of electronics and information technologies for a further automatization of production [3]. Nowadays, we are involved in the fourth Industrial Revolution, commonly called “Industry 4.0”, based on cyber-physical production systems (CPS) and embracing automation, data exchange and manufacturing technologies. These cyber-physical systems monitor the physical processes, make decentralized decisions and trigger actions, communicating and cooperating with each other and with humans in real time. This facilitates fundamental improvements to the industrial processes involved in manufacturing, engineering, material usage and supply chain and life cycle management [4].

Inside each revolution, several milestones have been achieved incrementing the innovation level. For instance, inside Industry 4.0, robot-based automatization has experimented its own revolutions. The first robotic revolution was the industrial automatization, the second one was the introduction of sensitive robots for safe automatization, the third one was the mobility with mobile manipulators, and the fourth and the last one is based on intelligent and perceptive robot systems [5].

The European Commission has set as objective for the Horizon 2020 Work Programme to achieve the leadership in industrial technologies (*i.e.*, Public-Private Partnership Factories of the Future or PPP FoF [6]). For this purpose, process automation and decreased accident rates are both important. Productivity and safety were limited by manual processes in the traditional industry; automatization and intelligent robots drive modern industry towards efficiency, resulting in a rapid increase in productivity, major material and energy savings and safer working conditions. Manufacturing demonstrates a huge potential to generate wealth and to create high-quality and highly skilled jobs [7]. In fact, several technology enablers have been identified within PPP FoF, such as advanced manufacturing processes and technologies, mechatronics for advanced manufacturing systems, including robotics, information and communication technologies (ICT), manufacturing strategies, knowledge-workers and modelling, simulation and forecasting methods and tools. Moreover, the European Commission has identified robotics as a key issue due to its importance for European economy: Europe is one of the world leading regions in industrial robotics with a share of more than 25% of supply and use. It is expected that robotics growth reaches 32 B$ by 2016 and a direct impact on job creation as forecasts determine that each industrial robot needs at least four people to run, maintain and service it [8]. Robotics directly improves society and better living conditions as it addresses global concerns such as climate change, sustainable transport, affordable renewable energy, food safety and security, and coping with an ageing population. Some examples of European projects in this area are TAPAS [9,10], VALERI [11,12] and SYMBIO-TIC [13].

Vision systems are widely used in industry, mainly for inspection and quality control processes [14]. Their use has been increased in applications related to improving the safety of workers in the industrial environment and for robot guidance [15]. Robots need machine vision to move around the working space avoiding obstacles, to work collaboratively with humans, to identify and locate the working parts, to improve their positioning accuracy, *etc*. Depending on the objective, the vision system can be scene-related or object-related [16]. In scene-related tasks the camera is usually mounted on a mobile robot and applied for mapping, localization and obstacle detection. In object-related tasks, the camera is usually attached to the end-effector of the robot manipulator (eye-in-hand configuration), so that new images can be acquired by changing the point of view of the camera.

Industrial robots are able to move to a position repeatedly with a small error of 0.1 mm, although their absolute accuracy can be several mm due to tolerances, eccentricities, elasticities, play, wear-out, load, temperature and insufficient knowledge of model parameters for the transformation from poses into robot axis angles [17,18,19]. In the automotive industry the accuracy requirement for operations such as spot welding will be of the order of 1 mm. The aerospace industry provides a challenging environment to apply robotics as its accuracy requirements are at least a factor of ten- to twenty-fold higher [20]. Conventional robots are not capable of achieving this accuracy. To improve the accuracy, optical calibration methods, such as laser tracker systems, photogrammetry or vision systems with multiple high resolution cameras, are used to detect the spatial position of the tool tip and to correct the robot motion. The combination of a measurement system with a robot is an optimal solution as it makes use of the robot ability for precise movement and overcomes the accuracy deficiencies. Moreover, working parts can be positioned slightly different from what the robot is expecting. In order to successfully navigate a mobile robot, obtaining detailed information on its immediate environment is the main concern. If the collected data is correct and sufficiently detailed, the creation of a 3D appearance model is possible and developers are given the opportunity to create accordingly sophisticated navigation software.

At this point, machine vision techniques and robotics become the main actors in the industrial scenarios. Thus, this work is a comparative review of different machine vision techniques and their applications for robot guidance, studying and analyzing the state of the art and the different approaches. The suitability of each vision technique depends on its final application, as requirements differ in terms of accuracy, range and weight of the sensors, safety for human workers, acquisition and processing time, environmental conditions, integration with other systems (mainly the robot), and budget. Main challenges are found in textureless surfaces for the correspondence problem, lighting conditions that may cause brightness, occlusions due to the camera point of view, undetermined moving objects, *etc*. Other comparatives can be found in the literature although they are oriented to commercial purposes, centered in one vision technique, or focused on software and algorithms. They are also analyzed in this work.

The rest of the paper is organized as follows: Section 2 resumes the fundamentals of vision-based 3D reconstruction, Section 3 reviews a wide range of robot guidance applications using machine vision, Section 4 performs a comparative analysis according to the application requirements and discusses advantages and drawbacks, and finally main conclusions are found in Section 5.

## 2. Fundamentals of 3D Reconstruction

Three-dimensional perception is one of the key technologies for robots. A 3D view of the surroundings of the robot is crucial for accomplishing navigation and manipulation tasks in a fully autonomous way in incompletely known environments. Moreover, tele-operation of robots requires a visualization of the environment in a human-readable way, which is important for an intuitive user interface. Thus, vision systems for robot guidance generally need to obtain 3D information.

**Figure 1:**
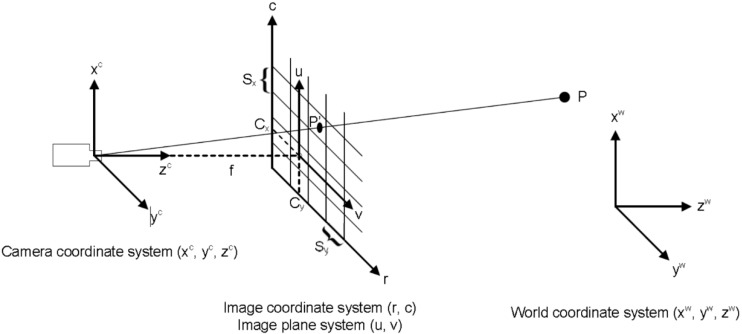
Pinhole camera model [23].

Given a point in the scene, its corresponding point in the image can be obtained by mathematical models [21]. This is the direct problem. As it determines a set of parameters that describe the mapping between 3D points in the world coordinate system and the 2D image coordinates, this process is also known as camera calibration. The perspective projection of the world coordinates onto the image is generally modeled using the pinhole camera model [22]. Figure 1 shows a graphical representation. Using this model, the image of a 3D point, *P*, is formed by an optical ray passing through the optical center and intersecting the image plane. The result is the point *P’* in the image plane, which is located at a distance *f* (focal length) behind the optical center.

The first step to mathematically describe the projection of 3D points on the 2D image plane is the transformation from the world coordinate system *W* to the camera coordinate system *C*. This transformation is given by Equation (1). Using this equation, the camera coordinates of a point *P_c_ = (x_c_, y_c_, z_c_)^T^* are calculated from its world coordinate *P_w_ = (x_w_, y_w_, z_w_)^T^* using the rigid transformation *H_w→c_*:

(1) $$\left(\begin{array}{c} P^{c}\\ 1 \end{array}\right)=H_{w\to c}\left(\begin{array}{c} P^{w}\\ 1 \end{array}\right)$$

The homogeneous transformation matrix *H_w→c_* includes three translations *(t_x_, t_y_, tz)* and three rotations *(α, β, γ)*. These six parameters are called the extrinsic camera parameters and describe the rotation (*R_w→c_*) and translation (*t_w→c_*) from *W* to *C*. Thus, Equation (1) can also be expressed as Equation (2):

(2) $$\left(\begin{array}{c} \begin{array}{c} x^{c}\\ y^{c} \end{array}\\ \begin{array}{c} z^{c}\\ 1 \end{array} \end{array}\right)=\left(\begin{array}{cc} \begin{array}{c} R_{w\to c}\\ \begin{array}{ccc} 0 & 0 & 0 \end{array} \end{array} & \begin{array}{c} t_{w\to c}\\ 1 \end{array} \end{array}\right)\left(\begin{array}{c} \begin{array}{c} x^{w}\\ y^{w} \end{array}\\ \begin{array}{c} z^{w}\\ 1 \end{array} \end{array}\right)$$

Based on the pinhole model, the projection of the point in the camera coordinate system *C* onto the image coordinate system is calculated using Equation (3):

(3) $$\left(\begin{array}{c} u\\ v \end{array}\right)=\frac{f}{z^{c}}\left(\begin{array}{c} x^{c}\\ y^{c} \end{array}\right)$$

The pinhole model is only an ideal approximation of the real camera projection. Imaging devices introduce a certain amount of nonlinear distortion [24]. Thus, when high accuracy is required, lens distortion must be taken into account [25]. The final step is the transformation from the image plane coordinate system *(u, v)* to the image coordinate system *(r, c)*, which is the pixel coordinate system. This transformation is achieved using Equation (4), where *S_x_* and *S_y_* are scaling factors that represent the horizontal and vertical distances between the sensor elements on the CCD chip of the camera and the point *(C_x_, C_y_)^T^*, which is the perpendicular projection of the optical center onto the image plane. Equation (4) reflects the calibration matrix:

(4) $$\left(\begin{array}{c} r\\ c \end{array}\right)=\left(\begin{array}{c} \frac{v}{S_{y}}+C_{y}\\ \frac{u}{S_{x}}+C_{x} \end{array}\right)$$

**Figure 2:**
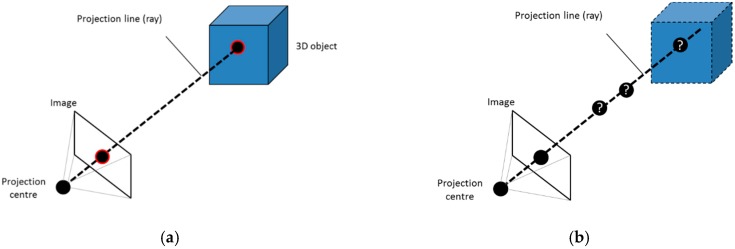
From 3D to 2D. (**a**) Direct problem; (**b**) Inverse problem.

The projection of a point in the scene on the image can be mathematically calculated as shown in Figure 2a. However, given a point of the image it is not possible to obtain directly its original point in the space, as it is not a one-to-one relationship, but one-to-several, thus the inverse problem is ill-defined [26]. In algebraic terms, the projection of a 3D point on the image is not an injective application. Different points can be projected on the same pixel. What is really obtained by solving the inverse problem is a straight line formed by all points that are represented on the same pixel of the image. This is the projection line shown in Figure 2b.

Passive techniques, such as stereo vision or photogrammetry, which only require ambient lighting, solve the problem by looking for the same point in multiple images and computing the intersection of the projection lines. Others project a visible or infrared pattern onto the scene and estimate the depth information from the returning time (time of flight), the deformation of the pattern (light coding) or trigonometric calculations (laser triangulation and structured light). They are active vision. This difference in illumination method is important since the less well-defined features an object may have, the less accurate the system will be when passive vision is used. This is not the case with active vision systems, since a known pattern is used to illuminate the object. Nevertheless, using active vision can result in measurement inaccuracies, especially in the edges or in objects with varying surface finishes [27]. The main disadvantage of non-contact measurement systems is their high sensitivity to various external factors inherent to the measurement process or the optical characteristics of the object [28]. Passive vision techniques need multiple cameras for 3D reconstruction and active ones only use a single camera. However, as it will be shown, some techniques can be passive or active vision or use one or several cameras depending on the application. In addition, some of them are in fact evolutions or improvements of others. Thus, depending on the authors different classifications can be found over the literature. Table 1 shows one possible classification and points out that some technique can be included in more than one group.

Numerous sensor technologies are available, and each of them provide unique advantages for its use in specific environments or situations. In the following subsections, different 3D vision techniques are presented.

### 2.1. Stereo Vision and Photogrammetry

Literally photogrammetry consists of measuring real dimensions from a photo of an object. It is a 3D reconstruction technique based on conventional 2D images commonly used in architecture [29,30,31,32], topography [33,34], geology [35], archaeology [36], engineering [37,38], and manufacturing [39].

**Figure 3:**
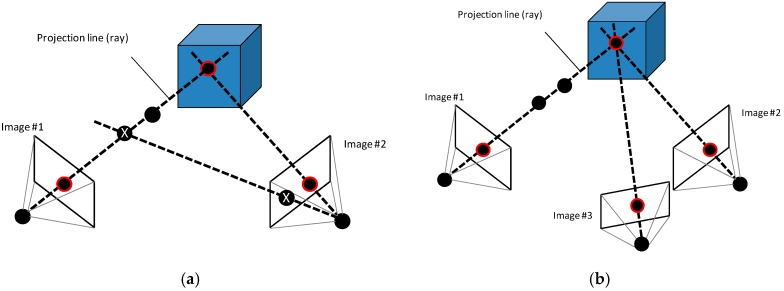
From 2D to 3D. (**a**) Homologous points; (**b**) Intersection of the projection lines.

In stereo vision and photogrammetric techniques, the same point has to be found in other image to calculate the intersection of the projection lines and to obtain the 3D position (Figure 3). However, it is recommended that every point could be found at least in three images in order to ensure the detection and to improve the accuracy. The selected points must be the homologous and not any other ones in order to get the right 3D position.

**Figure 4:**
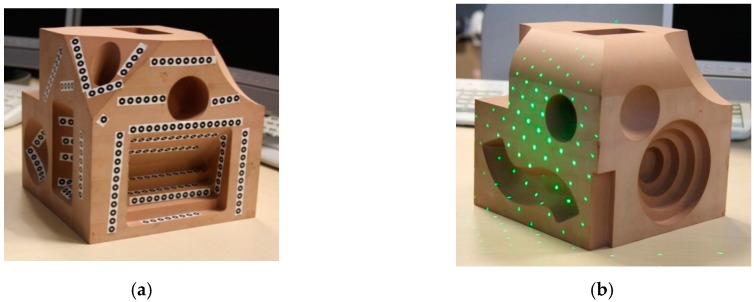
Physical marks used in marker-based stereo vision. (**a**) Stickers; (**b**) Laser points.

**Figure 5:**
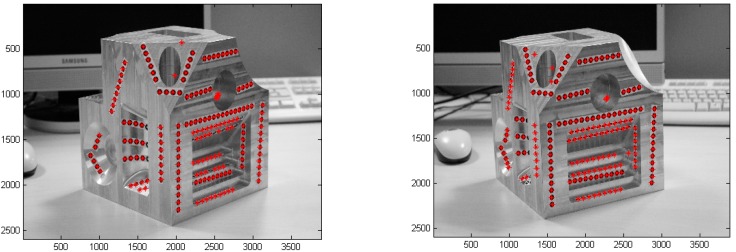
Detection of marks in several images.

Physical marks, such as stickers or laser points (Figure 4), are necessary over and around the object (the more, the better) and they must be of high contrast in order to ensure the detection. In Figure 5 two images of the same marked part have been taken and processed. The detected marks are printed in red. If the spatial position of the cameras and their calibration parameters are known, the marks can be paired using epipolar geometry and their projection lines and their intersections can be calculated to find the 3D position [40]. Notice that only those marked points are detected and used for the model (marker-based stereo vision).

**Figure 6:**
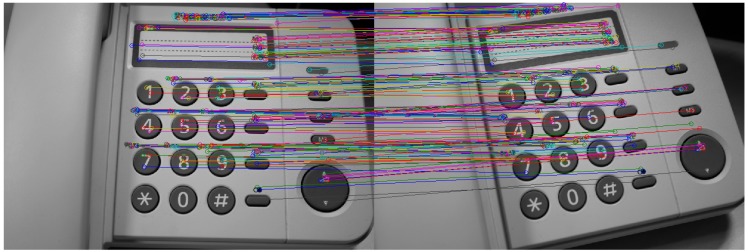
Feature tracking algorithms.

In some cases, it is desirable to avoid these physical marks in order to save time and to automate the process. Feature tracking algorithms find, extract and match intrinsic characteristics of the objects between similar or consecutive images- avoiding physical marks, as can be seen in Figure 6 (markerless stereo vision).

The extracted features depend on the problem or the type of application. In fact, a characteristic feature is a region of interest in the image that provides important information, such as an edge, a corner, bright or dark isolated points, *etc*. These detected points are useful for a subsequent search of correspondences between similar images. Some of the most popular algorithms for features detection, extraction and tracking are Canny [41], Harris [42], KLT [43], SIFT [44], SURF [45], and MSER [46].

Markerless stereo camera systems are widely used in many real applications including indoor and outdoor robotics. They provide accurate depth estimates on well-textured scenes, but often fail when the surface of the object is low-textured or textureless. In this case, it is necessary to project a known static high contrast light on it highlighting points, features, non-visible structures, *etc*. and creating an artificial texture. Then, the reflected light is captured using a stereo camera system and a matching algorithm associates the homologous points to obtain the 3D information [47]. *Ensenso* has developed several series of compact sensors based on this technique [48].

The projected texture is usually pulsed infrared light which is not affected by external light sources. It can take many forms including crosses, circles, squares, dot-matrices, multiple lines and random dot matrices. Finding the optimal texture, that is, the one which provides the best correspondence between features of the images, is a complicated problem, influenced by characteristics of the projector, the pattern, and the stereo cameras [49,50].

### 2.2. Time of Flight

**Figure 7:**
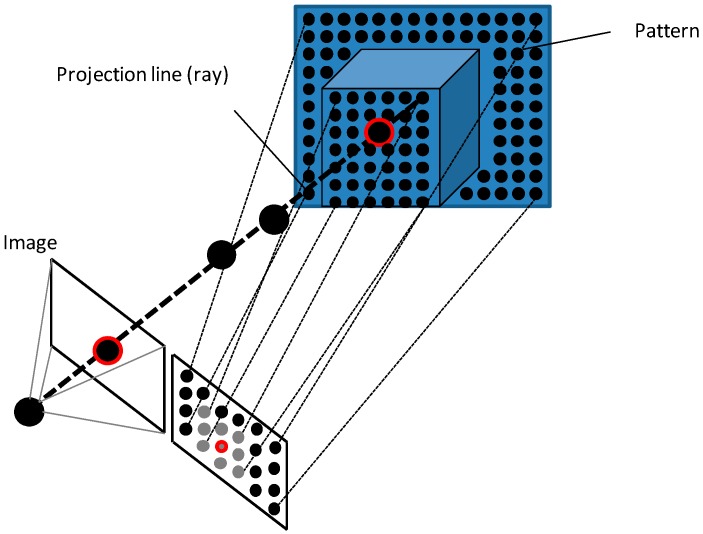
Projecting a pattern on the object.

Active vision techniques obtain the 3D information projecting a visible or infrared pattern on the object as shown in Figure 7. A time of flight (ToF) camera is a range camera that uses light pulses. The illumination is switched on for a very short time. The resulting light pulse is projected on the scene illuminating it and being reflected by the objects. The camera lens captures the reflected light onto the sensor plane. Depending on the distance, the incoming light experiences a delay which can be calculated as shown in Equation (5), where *t_D_* is the delay, *D* is the distance to the object and *c* is the speed of light. The pulse width of the illumination determines the maximum range the camera can handle, thus the illumination unit is a critical part of the system. Only with some special LEDs or lasers it is possible to generate such short pulses.

(5) $$t_{D}=2\cdot \frac{D}{c}$$

One of the most common sensors has been developed by *MESA Imaging* [51]. Although ToF sensors open new possibilities and applications as they are quite compact and lightweight, and allow real-time distance acquisition, the quality of raw data is quite noisy and prone to several types of disturbances. They involve major specific challenges: (1) Low resolution compared with other techniques; (2) Systematic distance error; (3) Intensity-related distance error, as the distance is influenced by the incident light; (4) Depth inhomogeneity mainly at object boundaries; (5) Motion artifacts leading to erroneous distance values; (6) Multiple reflections; and (7) Other general aspects of active systems [52].

### 2.3. Structured Light

Structured light equipment is composed of a light source (the light projector) and one or two information receptors (the cameras). Among all the structured light techniques, there are two main groups [53]: time-multiplexing techniques, which are based on projecting a sequence of binary or grey-scaled patterns, and one-shot techniques, which project a unique pattern. The advantage of time-multiplexing techniques is that, as the number of patterns is not restricted, a large resolution, *i.e.*, number of correspondences, can be achieved. However, their main constraint is that the object, the projector and the camera must all remain static during the projection of the patterns. In one-shot techniques a moving camera or projector can be considered. In order to concentrate the codification scheme in a unique pattern, each encoded point or line is uniquely identified by a local neighbourhood around it [54].

**Figure 8:**
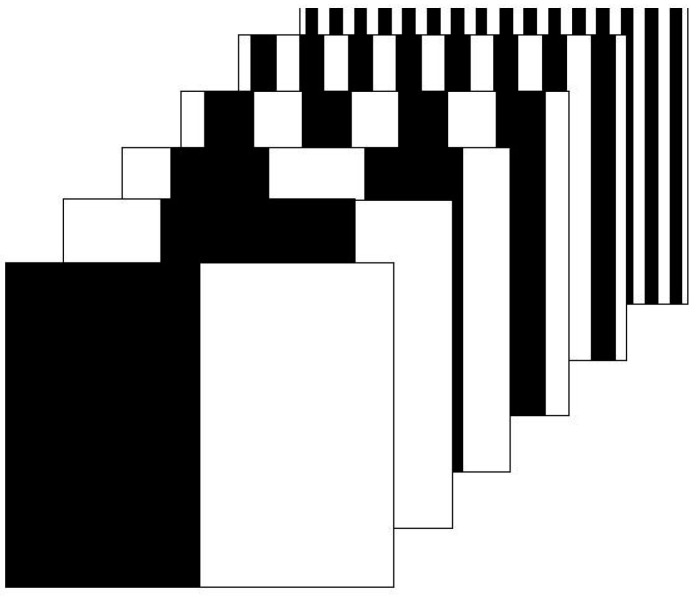
Structured light typical patterns.

Generally, the projected light is white light which is easily generated and is not dangerous for people unlike laser. This light is modified by grids to create lines or bands with lights and shadows like a zebra (Figure 8) which are recorded by the camera. Depth is obtained from the deviations using a technique similar to triangulation which consists on calculating the intersection between planes and lines [55].

**Figure 9:**
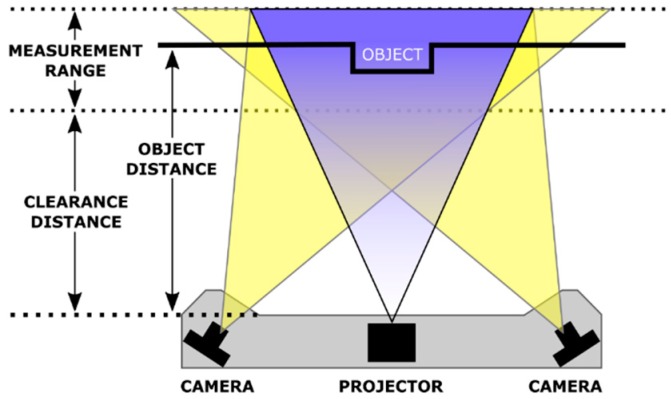
Blue LED sensor components.

Non-contact 3D digitizing scanners derived from structured light projection are increasingly more accurate, fastest and affordable [56]. Recently, new scanners have been launched based on blue light instead of white. As Figure 9 shows, they use a structured blue LED light module and a stereo scanner to generate the 3D point cloud. The LED module produces high contrast patterns allowing a high resolution scanning of the scenario. It is not affected by external light sources and it is safety for people. Sensors as the *HDI 109* and *HDI 120* from *LMI Technologies* can achieve an accuracy of 34 µm and 60 µm, respectively [57].

### 2.4. Light Coding

Recently a whole new type of sensor technology called light coding has become available for purchase at only a small fraction of the price of other 3D range finders. Light coding uses an entirely different approach where the light source is constantly turned on, greatly reducing the need for precision timing of the measurements. It can be considered an evolution of structured light.

**Figure 10:**
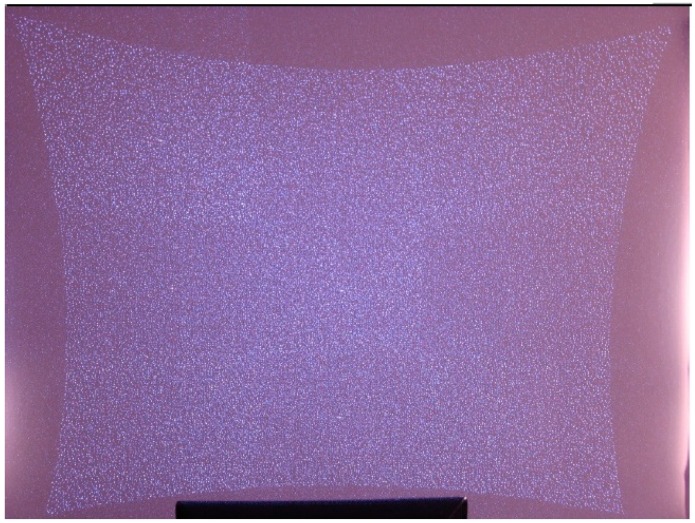
Projected pattern in Light coding.

A laser source emits invisible light (approximately at the infrared wavelength) which passes through a filter and is scattered into a semi-random but constant pattern of small dots which is shown in Figure 10. The reflected pattern is then detected by an infrared camera and analyzed. From knowledge on the emitted light pattern, lens distortion, and distance between emitter and receiver, the distance to each dot can be estimated measuring the deformations in shape and size of the projected points [58]. The technique has been developed by the company *PrimeSense* and its most commonly extended sensor is *Microsoft Kinect*.

Light coding offers depth data at a significantly low cost, which is a great innovation not only for robotics. However, it has some limitations as these cameras do not provide a dense depth map. The delivered depth images contain holes corresponding to the zones where the sensor has problems, whether due to the material of the objects (reflection, transparency, light absorption, *etc*.) or their position (out of range, with occlusions, *etc*.). The depth map is only valid for objects that are in the range of 1–3 m in order to reduce the effect of noise and low resolution [59]. In addition to this, as it is based on an IR projector with an IR camera, and as the sun emits in the IR spectrum, sunlight negatively affects it.

### 2.5. Laser Triangulation

**Figure 11:**
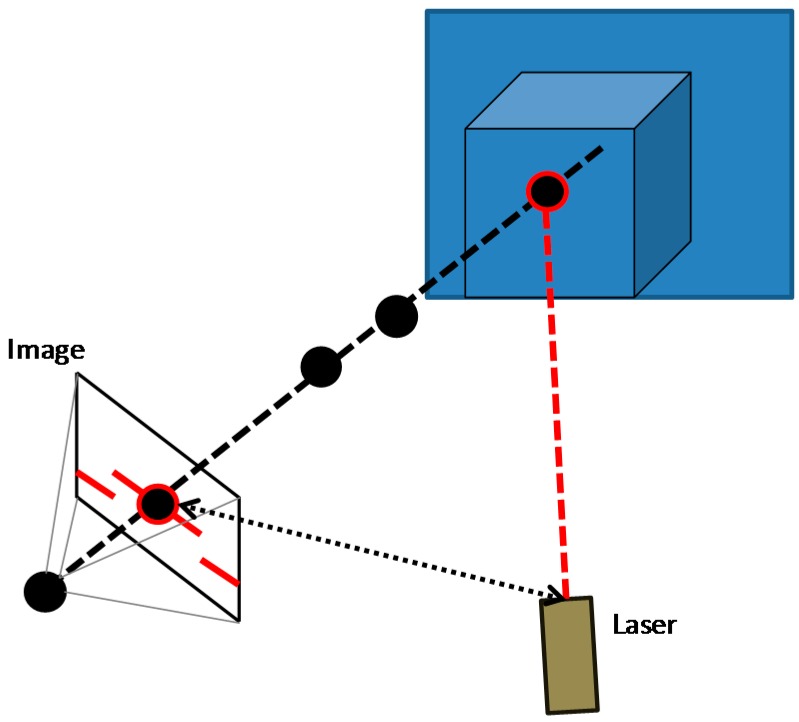
Laser triangulation.

In laser triangulation, the point, the camera and the laser emitter form a triangle (Figure 11). The distance between the camera and the laser emitter is known, and because of the angle of the laser emitter corner is also known, the angle of the camera corner can be determined by looking at the location of the laser dot in the camera's field of view. These three pieces of information fully determine the shape and the size of the triangle and give the location of the laser dot corner of the triangle, which is in fact the 3D point. The accuracy depends on the resolution of the CCD sensor, the quality of the lenses, the point size (spot), the laser beam quality, the surface state of the piece and other optical factors [60].

Laser non-contact techniques have changed considerably in recent years. Although initially sensors gave isolated points, the technology has quickly spread to 2D sensors, measuring across a dotted line and collecting multiple points in a single frame. If the piece is moving under the sensor (or *vice versa*), the 3D model of the surface can be generated. This means that, unlike the previously presented techniques where a 3D point cloud can be obtained with a single capture, in this case it is necessary to move the piece or the sensor. In other words, it is necessary to scan the piece. As a drawback, depending on the laser power, laser sensors can be dangerous for people. They are not eye-safe.

## 3. Robot Guidance in Industrial Environments

Robot guidance using machine vision techniques is a challenging problem as it consists on providing eyes to a machine which is able to move with high repeatability but low accuracy [18] in complex industrial environments with other moving objects or even human workers. This modern technology opens up wholly new possibilities although it also creates new and fairly complex challenges in safety design [61]. Textureless surfaces, lighting conditions, occlusions, undetermined or moving objects, among others, are critical issues which the vision system has to deal with. As shown, 3D point cloud acquisition for robot pose estimation, robot guidance or any other purpose can be achieved by applying many different sensors and techniques. Which one is best suited for each particular application really depends on the needs and requirements [57]. The spatial coordinates of a large number of points can be obtained almost instantaneously or in a few seconds, but they require further treatment. Point clouds must be processed using specific algorithms to identify the geometric elements of the parts to be detected, measured or positioned. Subsequently filtering operations, structuring or interactive segmentation of the point clouds must be carried out [62]. The quality and the robustness of the final application are determined by both processes: the point cloud acquisition and the subsequent treatment.

**Figure 12:**
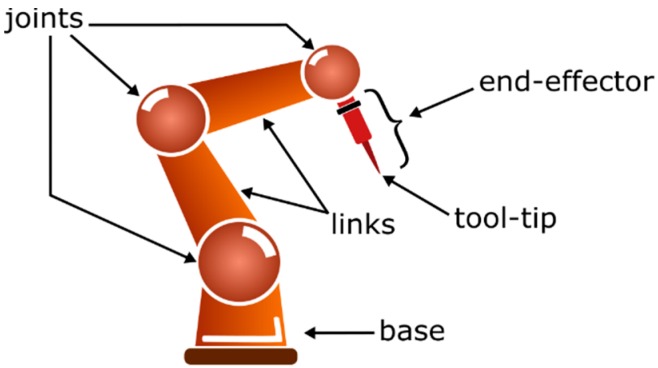
Robot terms.

In this section several approaches of robot guidance using different machine vision techniques are reviewed. As it will be shown, applications are oriented to scene-related tasks for environment reconstruction, including people detection, or to object-related tasks for robot pose estimation and object reconstruction for manipulation or inspection. Figure 12 provides the most common terms of robots that will be mentioned throughout the text.

### 3.1. Stereo Vision and Photogrammetry

Industrial photogrammetry covers different practical challenges in terms of specified accuracy, measurement speed, automation, process integration, cost-performance ratio, sensor integration and analysis. Main solutions are object-related in the fields of the measurement of discrete points, deformations and motions, 6 DOF (degrees of freedom) parameters, contours and surfaces [63]. Off-line photogrammetry systems can be found at automotive manufacturing (for car body deformation measurement, control of supplier parts, adjustment of tooling and rigs, *etc*.), the aerospace industry (for measurement and adjustment of mounting rigs, alignment between parts, *etc*.), wind energy systems (for deformation measurements and production control), and engineering and construction (for measurement of water dams, tanks, plant facilities, *etc*.). They offer high precise and accurate measurements (the absolute accuracy of length measurements is generally about 0.05 mm for a 2 m object [64]). On the other hand, on-line systems provide 3D information to control a connected process. Some examples include tactile probing (where a hand-held probing device is tracked in 3D space in order to provide the coordinates of the probing tip), robot calibration (where the robot tool center point is observed in order to determine its spatial trajectory), and sensor navigation (where a measurement device, such as a laser profile sensor, is tracked in 6 DOF in order to reconstruct the captured profiles). The accuracy of on-line systems is in the order of 0.2–0.5 mm over a range of 2 m [65], usually less than that of off-line systems. Nowadays, industrial photogrammetric systems are mostly used for off-line measurement of static 3D points in space. Moving from off-line to on-line systems is mainly a matter of speeding up image processing. Image acquisition, transfer, target identification and measurement usually take by far the largest part of the processing time.

**Figure 13:**
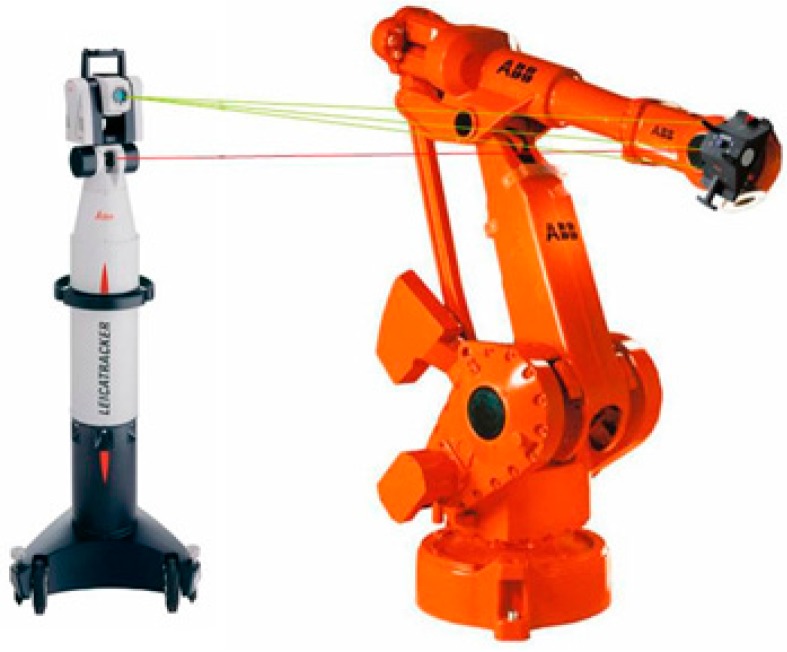
Laser tracker.

There are several approaches in the literature to determine the position and orientation of a robot's end-effector with high accuracy during arbitrary robot motions based on combined and pure photogrammetric solutions. Laser tracking systems combine laser interferometry and photogrammetry [66,67]. As Figure 13 shows, the end-effector of the robot (the probe) is equipped with a number of LED reference targets suitable for camera imaging, as well as a retro-reflector suitable for laser tracking, all with calibrated local coordinates. The 6-DOF pose of the probe is measured by space resection through a camera that is integrated into the laser tracker. Distance information is provided by interferometric laser ranging while the camera measures angular information of the probe [68]. Laser trackers are also used to identify the geometric and dynamic parameters of the robot in order to improve the accuracy of the model, increasing thus the accuracy of the robot [69]. Qu [70] presents a laser tracker application to reduce the relative pose error of the robot of an aircraft assembly drilling process to less than 0.2 mm.

In pure photogrammetric solutions, there are three different approaches [18]: (1) Forward intersection, where two or more fixed cameras that are observing target points which are mounted on the end-effector (moving targets); (2) Resection, where alternatively one or more cameras can be mounted on the end-effector observing fixed targets; and (3) Bundle adjustment, which is a combination of both. At a first sight, resection arrangement may seem to be inferior compared to the forward intersection method, because a pose measurement for all possible robot poses requires targets to be placed around the entire workspace. However, most handling tasks require a robot to be accurate only in certain locations, thus the accuracy is not needed in the entire workspace.

Main documented applications are for 6-DOF measurements, robot calibration, object tracking, and robot navigation. Several simultaneously operating solid-state cameras and photogrammetric hardware and software for robot guidance tasks are used in [71]. In this work two different applications were chosen to demonstrate the accuracy, flexibility and speed of the photogrammetric system: 3D object positioning is utilized in the measurement of car body points for accurate seam-sealing robot operation, and a robotized propeller grinding cell uses profile and thickness measuring data to control the grinding process. Hefele [18] shows an off-line photogrammetric robot calibration using a high resolution camera and targets mounted to the robot end-effector. The positioning accuracy of the robot is better than 3 mm in the experiments. First results towards an on-line photogrammetric robot tracking system are also presented in [18], reducing image processing and using intelligent cameras. The bundle adjustment indicates target coordinate RMS values of 0.06 mm in depth. Some improvements were added later in [72].

**Figure 14:**
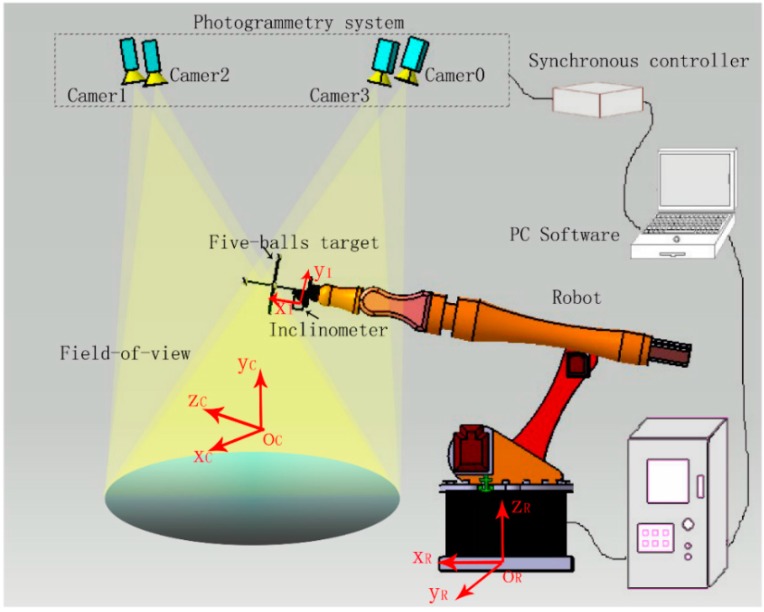
Multiple-sensor combination measuring system [73].

When accuracy requirements are moderate, [19] presents a digital photogrammetric system for industrial robot calibration tasks. Standard deviations of 0.05–0.25 mm in the three coordinate directions could be obtained over a robot work range of 1.7 × 1.5 × 1.0 m^3^. In this sense, [20] describes the development of a photogrammetric 6-DOF measurement system mounted on the end-effector of an industrial robot. The functionality of the system has been demonstrated for drilling and assembly operations showing that the scheme is feasible and assesses the capability of such a system to operate within realistic tolerances. The photogrammetric system shown in Figure 14 is proposed in [73] to improve the pose accuracy of industrial robots. Experimental results show that the position error of the robot manipulator is less than 1 mm after being calibrated by the photogrammetric system. Amdal [74] gives a description of an on-line system designed for close range applications. The system has the ability to perform 3D point measurements with one single camera in combination with a measurement tool equipped with photogrammetric targets, which are precalibrated in the local tool coordinate system. Accuracy results obtained from simulation studies and measurement tests are reported. For a camera-to-object distance of 2.5 m, the accuracy was found to be 0.01 mm.

Consequently, photogrammetry is certainly able to determine robot pose accurately [75] although these measurements require special and expensive equipment and processing huge amounts of image data. Because of these reasons, nowadays photogrammetry is mainly limited for calibration, which is performed only once during the setup of the robot at the factory, instead of a continuous tracking of the pose. Recent and future developments are concentrated on higher dynamic applications, integration of systems into production chains, multi-sensor solutions and lower costs.

**Figure 15:**
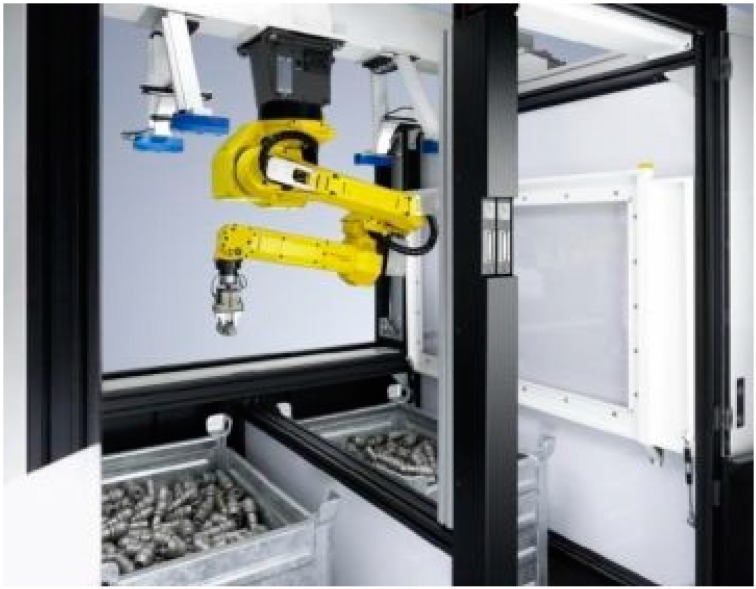
Bin picking [77].

Projected texture stereo vision technique is mainly used in bin picking applications [76,77]. The task of picking random and unsorted objects from a container or a storage bin presents a number of different challenges. A fast and reliable identification of one or several objects is required in terms of shape, size, position and alignment (Figure 15). This information must be obtained and passed on to the robot controller, which is essential to the *ad hoc* generation of collision-free robot paths. This is the starting point for the use of robots in handling processes.

Sturm [78] presents an approach for detecting, tracking, and learning 3D articulation models for doors and drawers using projected texture stereo vision system. The robot can use the generative models learned for the articulated objects to estimate their mechanism type, their current configuration, and to predict their opening trajectory.

### 3.2. Time of Flight

ToF sensors have several advantages for the development of robotic applications as they are quite compact and lightweight, and allow real-time 3D acquisition with high frame rate. They are used in scene-related tasks, generally involving mobile robots and large displacements, and in object-related tasks, involving instead robotic arms or humanoid-like robots and small depths. However, they involve some challenges as the quality of raw data is quite noisy. To overcome this limitation, some authors apply calibration methods to rectify the depth images in order to obtain better results. Others complement ToF camera information with color cameras to create a 3D point cloud with real features, with grayscale cameras for redundant stereo or with laser scanners.

In the field of scene-related task, ToF camera capabilities in terms of basic obstacle avoidance and local path-planning are evaluated in [79] and compared to the performance of a standard laser scanner. May [80] presents a new approach for on-line adaptation of different camera parameters to environment dynamics. These adaptations enable the usage reliably in real world (changing) environments and for different robotic specific tasks. In [81,82] it is proposed the use of surface normals to improve 3D maps for badly conditioned plane detection. Others, such as [83,84], cope with ToF noisy point clouds using the Iterative Closest Point algorithm to find the relation between two point clouds. Arbeiter [85] performed an environment reconstruction for a mobile robot combining a ToF camera with two color camera, which is the input for a modified fast-SLAM algorithm. This algorithm is capable of rendering environment maps. Kuhnert and Netramai [86,87] combined a ToF sensor and a stereo system for environment reconstruction.

For object-related tasks, ToF cameras have also been successfully used for object and surface reconstruction, where the range of distances is smaller. Depending on the field of view of the camera, multiple 3D point clouds need to be acquired and combined. In fact, the most common setup usually includes a ToF camera mounted on the end-effector of a robotic arm to do the captures. Point cloud registration is more critical in object modeling than in scene modeling. Even if the hand-eye system is precisely calibrated, the displacement given by the robot is usually not enough and the transformation between different point clouds has to be calculated. Some examples of object modeling and object reconstruction can be found in [88,89,90,91]. For object manipulation, unknown and unsorted objects have to be identified or categorized in order to be grasped. Generally, it is not necessary to completely reconstruct the object. Some examples are described in [92,93,94].

Finally, for human-machine interaction, ToF does not require any special background and it is a non-invasive technique, contrary to the widely extended use of special gloves, artificial marks, special attached devices, *etc*. Thus, it is commonly used in human activity recognition than other vision techniques as [95] points out. This work reviews the state of the art in the field of ToF cameras, their advantages, their limitations, and their main applications for scene-related tasks, object-related tasks, and tasks involving humans.

### 3.3. Structured Light

**Figure 16:**
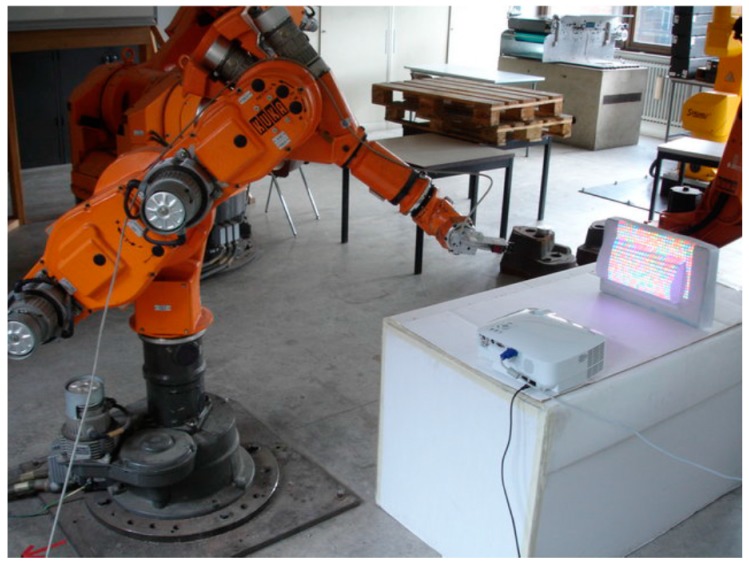
Robot positioning using structured light [96].

Positioning a robot with stereo vision depends on features visible from several points of view. Structured light provides artificial visual features independent of the scene, easing considerably the correspondence problem. The main inconvenience for robot guidance is the size of the sensors as they include a projector, which makes them difficult to be attached to the end-effector of a robot. Figure 16 presents a solution where only one camera is attached to the end-effector (eye-in-hand) and a static projector is installed illuminating the working pieces [96].

The achieved accuracy is 3 mm, which is enough for this concrete application. This setup also solves a problem related to the breaking of the hot lamp filament of the project due to vibrations if it is moved around. On the other side, an eye-in-hand setup avoids occlusions while it can perceive more details during robot approaching to the scene. The selection of the adequate pattern is also the main focus of most authors. Pagès [54] proposed a coded structured light approach as a reliable and fast way to solve the correspondence problem in another eye-in-hand solution with the projector aside the robot manipulator. In this case, a coded light pattern is projected providing robust visual features independently of the object appearance for robot positioning. Experiments have demonstrated that positioning the robot with respect to planar object provides good results even in presence of occlusions. Results when using non-planar objects show that the camera motion is noisier, slower and less monotonic.

Le Moigne [97] describes some of the important operational considerations and image processing tasks required to utilize a non-scanning structured-light range sensor in path planning for robot mobility. Particular emphasis is placed on issues related to operating in ambient lighting, smoothing of range texture, grid pattern selection, albedo normalization, grid extraction, and coarse registration of images to the projected grid. The created range map can be converted to a topography map for planning short-range paths through the environment while avoiding obstacles.

**Figure 17:**
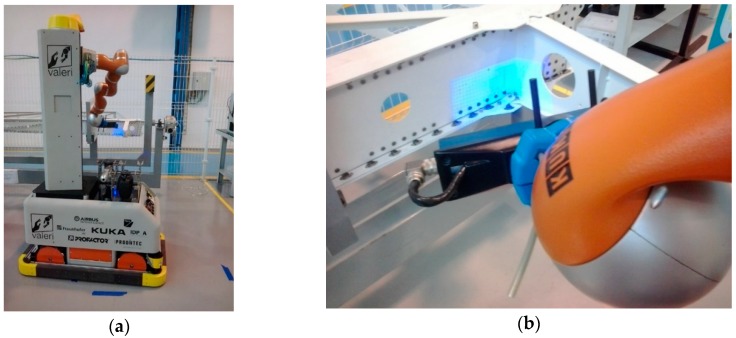
Mobile robot using a blue light sensor for part localization. (**a**) Mobile robot; (**b**) Sensor operating.

The new approach of structured blue light is not very extended yet in industrial robotic applications, but it is already being used for part identification and localization in [11], where a mobile and collaborative robot has been developed for aerospace manufacturing industries. Once the robot has reached the working station, it takes the camera, acquires images to get a point cloud (Figure 17) and compares it with a CAD databank, in order to identify the part and its pose. The robot also corrects its own pose to start with the assigned task (apply sealant or do a quality inspection). In this case, the point cloud is highly accurate. However, as the sensor working distance is too short, the robot has to be very close to the part and the scanned area is small. For small parts this is not a problem, but for long ones it requires an accurate CAD matching algorithm in order to avoid deviations in the robot trajectory.

### 3.4. Light Coding

Nowadays this technique is commonly used in videogames for people tracking. Besides, it is been introduced in more and more industrial applications including robotics although an extra effort is necessary to achieve accuracy for robot pose estimation, and safety requirements for workspace monitoring in human-robot collaboration. It offers visual and depth data at a significantly low cost and, while it is a great innovation for robotics, it has some limitations. Algorithms use depth information for object recognition, but as the sensor produces a noisy point cloud, it is required to improve such information. One possible option is the combination of light coding sensors with HD cameras to obtain high resolution dense point cloud which can be used for robot guidance or pose correction. Experimental results reported in [98] show that this approach can significantly enhance the resolution of the point cloud on both indoor and outdoor scenes. The point cloud is at least ten times denser than the initial one only with the light coding sensor.

**Figure 18:**
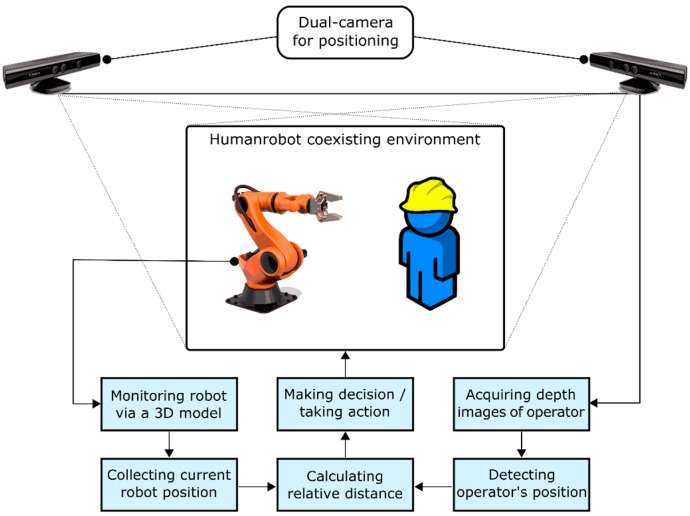
Combining 3D models of robots with information from sensors [100].

To significantly improve the robustness of people detection on mobile robots, light coding cameras have been combined with thermal sensors and mounted on the top of a mobile platform in [99], since humans have a distinctive thermal profile compared to non-living objects. This approach also combines classifiers created using supervised learning. Experimental results of this work have shown that the false positive rate (exclusively achieved using only the light coding sensor) is drastically reduced. As a drawback, some phantom detections near heat sources, such as industrial machines or radiators, may appear. Light coding sensors are also combined with safety certificated 3D zone monitoring cameras in [61]. Wang [100] combined virtual 3D models of robots with the information from the sensor (images of operators) for monitoring and collision detection (Figure 18). 3D models, which are used to represent a structured shop-floor environment and linked to real motion sensors, are driven to mimic the behavior of the real environment. Light coding sensors add unstructured foreign objects, including mobile operators, which are not present in the 3D models.

One common problem of light coding sensors is that they do not provide a dense depth map. The delivered depth images contain holes corresponding to the zones where the sensor has problems, whether due to the material of the objects (reflection, transparency, light absorption) or their position (out of range, with occlusions).

### 3.5. Laser Triangulation

This technique has been used in bin picking applications to pick up pieces, where it is necessary to recognize the piece and its pose. As it is necessary to scan the piece for 3D reconstruction, pieces are over a conveyor belt in [101] with a static camera and a static laser or camera and laser are integrated in robot tool for reconstruction and measurement as in [102]. Experimental evaluations of different line extraction algorithms on laser scans for mobile robots in indoor environments are presented in [103,104]. The comparison is carried out in terms of complexity, correctness and precision.

An implementation of a flexible, sensory-controlled robotic welding system is presented in [105]. Conventional, non-adaptive, robot welding systems can only be used when the workpieces are highly repeatable and well fixtured. A steerable cone of laser light and machine vision are used for sensing of the weld joint location and determining the detailed 3D weld joint surface geometry ahead of the welding torch. Robust vision-processing schemes for the detection and recognition of laser stripe features in noisy images are developed and implemented using a pipelined processing architecture. Approaches are proposed and implemented to incorporate the visually determined offsets in robot path planning and to control the welding process parameters. Another example of weld tracking is presented in [106]. This work offers a low-cost system that guarantees satisfactory tracking results even when the welding gap geometry varies strongly.

In [107] a sensor is created by coupling a camera and a laser stripe-. Positioning robotics tasks can be performed with good results, robustness and stability. Nevertheless, there are some constraints such as some restrictions in the laser stripe projection on to the scene. It is necessary to choose the most favorable location of the laser stripe during the calibration to achieve a robotics task under conditions of optimum stability. Pears [108] described a wide field of view range sensor for short range mobile robots maneuvers with an accuracy of 0.15% at l m, 1.3% at 2 m, and 3% at 2.5 m, and an average projected power of 0.9 mW, which is eye-safe. Generally, in robotics applications, it is necessary to take into account safety issues as human workers may be present. Depending on the laser power the sensor can result inappropriate for humans because of safety reasons.

Other works about robot navigation using laser scanners are described in [109,110,111]. In [109] the problems of self-localization of a mobile robot and map building in an *a priori* unknown indoor environment are addressed. In [110] a method for tracking the pose of a mobile robot using a laser scanner is presented. A new scheme for map building is proposed in [111]. This work describes localization techniques for a mobile robot equipped with a laser rangefinder using line segments as the basic element to build the map. According to the results, line segments provide considerable geometric information about the scene and can be used for accurate and fast localization.

## 4. Discussion

Depending on the final goal of the application and the type of robot, different considerations and factors need to be taken into account in order to select the most adequate vision technique:

- *Accuracy* of point cloud alignment and *resolution*. They are mainly determined by hardware (sensor) and software (extraction, registration, segmentation, comparison, *etc*.), and in consistence to the size of the object and the application purpose.
- *Range of the sensor*. The working distance will be determined by the accessibility of robot, size of sensor and environment configurations
- *Light weight*. If the sensor is onboard or mounted in the end-effector, the robot has limited max load weight to ensure its full dynamics.
- *Safety issues*. The robot might work closely with human workers, thus sensors should avoid dangerous high-power laser to minimize any risk of accidents.
- *Processing time*. Processing time might be crucial to determine if a system is suitable for a certain application, especially regarding moving robots with safety constraints, *i.e.*, availability to detect and avoid collisions with humans or obstacles. Some techniques require that object and camera remain static for the capture, thus they are not applicable for moving scenarios.
- *Scanning environment*. Lighting conditions, vibrations, camera movements, *etc*. can disturb the quality of the 3D point cloud in some techniques. It is necessary to avoid these interferences.
- *Hardware and software integration* with other systems. The camera will be automatically controlled by the own robot central control unit or by an external source. *Ad hoc* developments are oriented towards the integration and, nowadays, most of current commercial vision systems are also prepared to be connected to a robot and controlled by external software using SDKs or libraries.
- *Budget*. Outside of technical issues, for a real implementation, budget should also be considered. A trade-off between cost and performance is necessary as most of the previous characteristics can be achieved or improved incrementing the invested amount of money.

A comparison of vision techniques is presented in Table 2 in terms of accuracy, range, weight, safety, processing time, and scanning environmental influences (Env. influences). Some quantitative information is provided according to the referenced specific application. Stereo vision, structured light, and laser triangulation can provide acceptable accuracy under certain conditions for most applications. Except structured white light, active vision techniques need to be closer to the object as they have a short working distance. Nowadays, there are light weight commercial sensors available to be mounted on a robot. Structured white light sensors are in general the biggest. All techniques are not dangerous for people, with the exception of some high power lasers that are not eye-safe. In terms of processing time, photogrammetry requires processing a large amount of images, and structured light techniques require that object and camera remain static during the acquisition process. Time of flight and structured blue LED light are not influenced by environmental lighting conditions.

Table 3 compares advantages and disadvantages of the reviewed techniques for robot guidance. Photogrammetry is mainly used in static applications because of its accuracy, but physical marks, such as stickers or laser points, are necessary (marker-based) and it is highly influenced by brightness and lights in industrial environments. Marks can be avoided using feature trackers (markerless), but the density of the point cloud would be low if surfaces are textureless. In fact, low-textured or textureless surfaces are also an inconvenience for stereo vision techniques with conventional 2D cameras and it is necessary to project a high contrast light creating an artificial texture to highlight points, features, *etc*. (projected texture stereo vision). Other 3D active vision techniques, such as light coding and time of flight, have low theoretical accuracy and are not valid for certain applications where the point cloud is compared with a CAD model for accurate part localization, because flat surfaces are represented with rather significant curvature. They can be used for part identification or for people and object tracking considering these accuracy issues. Laser techniques are commonly used in scanning applications where the capture is not a single snapshot, but can be dangerous for people as some laser classes (high power) are not eye-safe. Finally, structured light provides accuracy, although sometimes is influenced by ambient light and has problems to create the 3D model for surfaces of certain colors. Its main disadvantage is that most commercial sensors are quite big to be carried by a robot. Actually, research and development efforts are concentrated on miniaturizing sensors or on separating the projector and the sensor so that only one is onboard the robot. In this sense, the new evolution of this technique called structured blue LED light provides accuracy with a small sensor.

There are thousands of industrial applications but most of them are confidential for companies and are not documented or widely described in scientific papers, thus they could not be included in this survey. Table 4 summarizes the references of vision techniques for robotics reviewed in this work grouped by scene-related and object-related tasks. According to the survey, main stereo vision applications in robotics are in the field of object-related tasks for robot pose estimation and robot calibration as they may require marks, camera and object must remain static and an important amount of information needs to be processed. Time of flight, which does not require that object and camera remain static, is mainly used for environment and object reconstruction, navigation, obstacle avoidance and indeed people detection. In fact, the main application of light coding is people detection, although it is not certificated for industrial environments. Laser-based sensors are widely used especially for navigation, but also for object-related tasks.

Some other comparative reviews of machine vision techniques for robotic applications can be found in the literature. Wilson [27] reviews 3D vision techniques and solutions in a journal article with commercial purposes providing a list of applications and manufacturers. Sets of questions and advises are presented in [57] in order to help the reader to choose the right 3D vision technique for his/her project. Some of these questions are relative to the size and the surface of the target object, to the accuracy requirements, to the final destination of the obtained 3D data, or to the budget. This classification is quite similar to the one proposed by the authors of this work in Table 2.

Foix [95] focuses on ToF cameras, describing advantages, limitations and applications. It includes 68 references grouped in scene-related, object-related and human-related. Visual human-machine interaction is deeply studied as ToF cameras can be used for gesture recognition. Focusing on software, [113] reviews vision-based control algorithms for robot manipulators and [114] analyzes stereo matching algorithms used in robotics in terms of speed, accuracy, coverage, time consumption, and disparity range. Implementations of stereo matching algorithms in hardware for real-time applications are also discussed.

Robots need flexibility and accuracy to carry out more complex and diverse tasks, such as collision avoidance with static and moving objects during navigation, collaborative work with humans, fine positioning, inspection, *etc*. In all the reviewed applications, each vision system has its single purpose. There has not been found one single vision system able to perform several tasks. Multiple vision systems are used (one for each individual purpose) instead of one single multi-tasking vision system. This is because requirements of each task are quite different and each technique has its scope and is more adequate than others.

## 5. Conclusions

In this survey, 3D vision solutions used in robotics have been organized, classified and reviewed. The purpose was to provide a compilation of the state of the art and the existing techniques so that future researchers and developers have a background information. Vision techniques for robot guidance have been analysed in terms of accuracy, range, weight, safety, processing time, and scanning environmental influences. Main advantages and main drawbacks have been also presented for each of them. Choosing which type of 3D vision system to use is highly dependent on the parts that need to be located or measured. While laser range finders using time of flight methods can be used to locate distant objects, stereo imaging systems may be better suited to imaging high-contrast objects. Where such objects are highly specular or textureless, it may be more useful to employ projected texture techniques. In addition to this, robot and industrial environments conditions also need to be considered. Each application and each type of robot need a specific vision solution. There is no universal vision technique to perform several tasks. Future woks may focus on multi-tasking or multi-purpose vision systems and their integration with other sensor types and systems.

Robots have become a core element of Industry 4.0 and flexibility can be incorporated to them by vision systems and other sensor technologies in order to achieve the requirements and functionalities of the new applications. New tasks are becoming more or more complex and it is necessary to improve the accuracy and to work collaborative with humans, which means making decisions in real-time and triggering actions. For these goals, visual feedback is the key issue, and this is in fact what vision systems provide to robots. Thus, 3D machine vision is the future for robotics. The idea of considering robot technology as an integral part of production is not new but nevertheless it is a challenge. Whether robots will be able to or should perform all the production steps in future is perhaps less a question of time than of money.

## Figures and Tables

**Table 1:** Vision techniques classification.

	Single Camera	Multiple Cameras
Passive vision	2D	Stereo vision Photogrammetry
Active vision	Time of flight Structured light Light coding Laser triangulation	Structured light Projected texture stereo vision

**Table 2:** Comparison of vision techniques in terms of accuracy, range, weight, safety, processing time, and environmental influences.

	Accuracy	Range	Weight	Safety	Processing Time	Environmental Influences
Stereo Vision and Photogrammetry	✓ (50 µm [64])	✓	✓	✓	✗ (image processing)	✗ (brightness)
Projected Texture Stereo Vision	✓ (0.1 mm [48])	✗ (0.25–3 m [48])	✓	✓	✗ (image processing)	✗ (brightness)
Time of Flight	✗ (10 mm [51])	✗ (0.8–8 m [16])	✓	✓	✓	✓
Structured White Light	✓ (0.127 mm [112])	✓	✗ (projector [96])	✓	✗ (remain static)	✗ (light, brightness)
Structured Blue LED Light	✓ (34 µm [57])	✗ (157–480 mm [57])	✓	✓	✗ (remain static)	✓
Light Coding	✗ (10 mm [58])	✗ (1–3 m [59])	✓	✓	✓	✗ (sun)
Laser Triangulation	✓	✓	✓	✗ (laser power)	✓	✗ (brightness)

**Table 3:** Advantages and disadvantages.

	Advantages	Disadvantages
Stereo Vision and Photogrammetry	Commonly used. Accuracy.	Influenced by environment. Physical marks are necessary. The density of the point cloud can be low. Object and camera must be static for the capture.
Projected Texture Stereo Vision	Physical marks are not required.	Influenced by environment. Object and camera must be static for the capture.
Time of Flight	Independent of ambient light. Not necessary that object and camera remain static for the capture.	Low theoretical accuracy.
Structured White Light	Accuracy.	Sometimes influenced by ambient light. Problems to create the 3D model for surfaces of certain colors. Expensive. Sensors can be quite large. Object and camera must be static for the capture.
Structured Blue LED Light	Accuracy. Small sensor.	Short working distance. Object and camera must be static for the capture. Expensive.
Light Coding	Inexpensive. Not necessary that object and camera remain static for the capture.	Low accuracy. Uncertified at industrial level.
Laser Triangulation	Commonly used. Inexpensive (depending on the laser, the accuracy).	Dangerous for people depending on laser power. Usually short working distance. Line scanner.

**Table 4:** Common applications in robotics.

	**Scene-Related**		**Object-Related**		
	People detection	Environment reconstruction / navigation	Object reconstruction / inspection	Bin picking / object manipulation	Robot pose / calibration
Stereo Vision and Photogrammetry			[74]		[18,19,20,67,68,69,70,71,72,73]
Projected Texture Stereo Vision				[76,77,78]	
Time of Flight	[95]	[79,80,81,82,83,84,85,86,87]	[88,89,90,91]	[92,93,94]	
Structured Light					[11,54,96]
Light Coding	[61,98,99,100]				
Laser Triangulation		[103,104,105,106,107,108,109,110,111]	[102]	[101]	[107]

## Abbreviations

The following abbreviations are used in this manuscript:

### Abbreviations

3D: Three-Dimensional
CPS: Cyber-physical Production Systems
PPP: Public-Private Partnership
FoF: Factories of the Future
ICT: Information and Communication Technologies
CCD: Charge-Coupled Device
2D: Two-Dimensional
CMM: Coordinates Measuring Machine
KLT: Kanade-Lucas-Tomasi
SIFT: Scale-Invariant Feature Transform
SURF: Speeded Up Robust Features
MSER: Maximally Stable Extremal Regions
ToF: Time of Flight
LED: Light Emitting Diode
IR: Infra-Red
DOF: Degrees Of Freedom
RMS: Root Mean Square
SLAM: Simultaneous Localization and Mapping
CAD: Computer-Aided Design
HD: High Definition
